# Taxonomic and Functional Response of Millipedes (Diplopoda) to Urban Soil Disturbance in a Metropolitan Area

**DOI:** 10.3390/insects11010025

**Published:** 2019-12-29

**Authors:** Zsolt Tóth, Elisabeth Hornung

**Affiliations:** Department of Ecology, Institute for Biology, University of Veterinary Medicine, Rottenbiller str. 50., H-1077 Budapest, Hungary; elisabeth.hornung@gmail.com

**Keywords:** Central Europe, diversity, functional trait, species richness, species turnover

## Abstract

Urbanization, as a major cause of local species extinction and biotic homogenization, drastically alters soil life. Millipedes are a key group of soil macrodetritivores and significantly influence soil quality, mainly through their essential role in nutrient cycling. Therefore, studying their taxonomic and functional responses to urban disturbance is crucial, as they contribute to the provision of several soil-related ecosystem services in cities. Differently degraded rural, urban forests and other woody patches (e.g., parks, gardens, and cemeteries) were sampled on Buda and Pest sides of the Budapest metropolitan area divided by the Danube River. We measured the most relevant physical and chemical properties of topsoil to characterize habitats. We applied an urbanization index based on vegetation cover and built-up area of the study sites to quantify urban intensity. The composition of the assemblages was determined by the division of the city along the Danube. Urbanization was associated with a reduction in species and functional richness of millipedes on both sides of Budapest. β diversity and species turnover increased with urban intensity. Urban disturbance was the main driver in assembly of taxonomic and functional community composition. A new species (*Cylindroiulus caeruleocinctus* (Wood, 1864)) to the fauna of Budapest was found. Detritivore invertebrates depend on leaf litter and other dead organic matter types, therefore microsites providing these resources greatly improve their survival. Due to increasing urban disturbance, it is recommended to provide appropriate detritus and shelter sites as part of the management of green spaces in order to maintain species richness, abundance, and function of species.

## 1. Introduction

Urban land-use is one of the most dominant forms of human activities in the world [1]. Urban areas continue to expand, causing anthropogenic disturbance of remaining natural habitats in a variety of ways; for example: Environmental pollution, habitat fragmentation, soil sealing and dispersion of exotic species, etc. Consequently, urban landscapes are generally characterized by destruction and a gradual replacement of native biota [2].

Urbanization, although causing local extinctions, does not always lead to a reduction in species richness, but rather to a shift in species composition, resulting in biotic homogenization of natural communities [3]. The idea that regardless of the “natural” and initial conditions, humans create similar landscapes in cities globally has been articulated in the “Urban Ecosystem Convergence Hypothesis” [4]. Homogenization of urban communities has been proven for several taxa, such as plants [5], microbes [6], insect orders [7,8] and birds [9]. However, soil macrodetrtivores, including millipedes, are heavily underrepresented in existing studies. 

The majority of urban studies have focused only on taxonomic α diversity (particularly on species richness) [3]. However, measuring β diversity, based on between-site differences, is more effective in identifying factors shaping community structure. Mechanisms influencing community assembly act on biotic complementarities and redundancies among sites [10,11]. Biotic complementary means that species have niches with no overlap, while biotic redundancy refers to niche overlap. A low β diversity is usually related to low heterogeneity in habitats, climatic conditions, or topography due to (anthropogenically driven) homogenization of these factors [12], thus making it highly relevant to understanding urban effects on biological communities. It is increasingly recognized that functional aspects of diversity are more directly linked to ecosystem functionality than taxonomic identity of species (e.g., [13]). Functional traits and their associated diversity indicate biotic interactions, niche complementarity, and environmental filtering [10]. It has been shown that better predictions of biological responses to habitat change may be gained from functional traits rather than taxonomic ones [14]. To understand the functionality of urban ecosystems better, an integrative approach is needed considering taxonomic and functional diversity at the same time. This is particularly important due to the significant role urban ecosystems play in providing ecosystem services with socio-economic benefits [15]. Although many ecosystem services are related to soils, soils and their biota have received little attention in urban context. Urban soils, like other soils, provide important ecosystem services, e.g., the maintenance of soil fertility and structural properties, filtering and providing a reservoir for water, nutrient cycling, and climate regulation [15]. Organic matter decomposition, as the most common ecosystem process in biogeochemical cycles, has an essential role in maintaining soil functions and quality [15].

Soil macrodetritivores significantly contribute to litter breakdown through their feeding and burrowing activities, thereby affecting organic matter level in soils [16]. Since millipedes (Diplopoda) are considered to be a key taxon in these ecological processes (particularly in temperate climates) [17], studying the diversity and composition of their assemblages is crucial. Millipedes are highly sensitive to disturbances and environmental changes, so they can be used as ecological indicators [18]. They are widespread, with limited dispersal abilities, and are relatively easy to collect and identify. It is well-known that microclimatic and edaphic factors represent strong environmental influences on the distribution of millipedes [19]. The most important soil characteristics for millipedes are soil texture, soil moisture content, temperature, mineral content (especially calcium and magnesium), and humus type [19,20].

Due to the two biogeographically distinct parts of Budapest with differing biological and physicochemical conditions, this city offers an ideal situation for studying the effects of urbanization, including generality/specificity and biotic homogenization, on millipede assemblages. 

Our aims were:

(1) To assess and compare millipede assemblages on the two sides of the city divided by the river Danube as a barrier;
(2) To investigate the taxonomic and functional diversity, and compositional response of millipedes to urban intensity;
(3) To identify the major soil properties shaping the structure of taxonomic and functional communities.

## 2. Materials and Methods

### 2.1. Study Sites and Design

The study was conducted in Budapest metropolitan area in Hungary. Budapest is the capital of Hungary and has about 1,752,000 inhabitants [21]. The city is divided by the Danube River, which separates the two major parts of the city, Buda and Pest. Buda (on the east bank of the river) can be characterized by a uniform parent rock, primarily limestone and dolomite, which forms a series of hills and valleys. The area included in this study is in the urbanized area of the Buda Hills, ranging from 300–500 m a.s.l. In contrast, Pest (on the west bank of the river) is mainly flat, generally 100–150 m a.s.l. and comprises the Danube floodplain, which overlies Triassic deposits. Their particular geological history led to very different landscapes with specific edaphic, botanical, and zoological characteristics [22]. Buda and Pest belong to the Matricum (Pilisicum sub-district) and Pannonicum (Eupannonicum sub-district) fauna districts, respectively [23]. 

**Figure 1:**
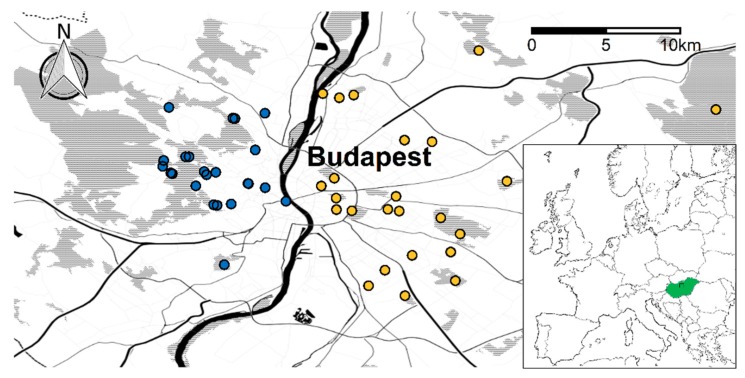
Locations of the sampling sites on the Buda (*N* = 23, blue dots) and Pest sides (*N* = 24, yellow dots) of Budapest. Inset: Map of Europe showing the position of Hungary and Budapest.

For site selection, we aimed at covering a wide spectrum of typical urban woody habitats. We selected 23 sites in Buda and 24 sites in Pest (Figure 1) with differing levels of disturbance and degradation: Rural forests, urban forests, and other woody patches (e.g., city parks, gardens, cemeteries). Rural forests are semi-natural woodlands, situated in nature reserves surrounding the city.

### 2.2. Diplopoda

#### 2.2.1. Sampling and Species Identification

Millipedes were collected by time-restricted hand sorting (60 min per site) [24] during their main activity seasons (spring and autumn) in 2016 (Buda side) and in 2018 (Pest side). During sampling, special attention was paid to discover favorable microhabitats, such as leaf litter, fallen tree trunks or branches, and shelter sites under barks and stones in order to maximize species richness. Individuals were preserved in 70% ethanol. The specimens were identified to species level using the keys of Schubart [25] and Korsós [26].

#### 2.2.2. Taxonomic and Functional Diversity

We assessed taxonomic and functional diversity of millipede assemblages for each individual site. Taxonomic α diversity was measured by species richness. To describe β diversity, pairwise comparisons between all sites were computed using the Jaccard dissimilarity index. This index enables partitioning of β diversity into the dissimilarity components due to species turnover (species replacement between communities) and nestedness (species loss or gain between communities) [27]. This method provides a more comprehensive picture of biotic homogenization, as the two components refer to different processes of community assembly [28]. The “beta.pair” function of the “betapart” R package was applied for calculation of the above-mentioned dissimilarity values [29].

Morphology (length and width) and ecological preference (habitat affinity, humidity preference, and disturbance sensitivity) of millipedes were used to characterize the functional structure and composition of assemblages (Table 1). Trait and preference values were based on a literature review (for values and literature sources, see Table S1, Supplementary Materials). Functional dispersion (FDis) index, as a representative of functional diversity, and community-weighted mean (CWM) trait score values were calculated using the “FD” R package [30]. FDis is the mean distance of species to the centroid of trait distribution. CWM represents the average of each trait/preference weighted by relative abundance/frequency [31].

### 2.3. Environmental Factors

#### 2.3.1. Landscape Structure Characteristics

An urbanization index (UI) was applied to quantify urbanization intensity, as proposed by Liker et al. [32]. Vegetation cover, building density, and the presence of sealed surface (roads) were scored for 100 cells of a 400 × 400 m area around each study site using the QGIS software (version: 2.16). For each site, urbanization index was calculated by extracting the first principal component (PCA1) from a principal component analysis (PCA) of five urbanization variables (mean building density, number of cells with high building density, number of cells with road, mean vegetation density, number of cells with high vegetation density). 

#### 2.3.2. Soil Properties

Composite samples consisting of 10 subsamples were taken randomly from the 0–15 cm topsoil layer of each study site. Soil pH (H_2_O) was measured in 1:2.5 soil/water suspensions for 12 h after mixing. Soil organic matter (SOM) was determined by the standard ignition method [33]. The total soluble salt content of soils was measured with a conductometer (Radelkis OK-102/1). To characterize soil texture, saturation percentage (SP), referring to the mechanical constituents of soils (as a proxy of soil plasticity), was applied [34]. Soil CaCO_3_ was determined with a Labor MIM calcimeter [35].

### 2.4. Statistical Analyses

All statistical analyses were performed in R software version 3.2.5., using the R packages “lme4” [36], “mvabund” [37], “nparcomp” [38], and “vegan” [39].

Principal component analysis (PCA) was used to compare the two sides of Budapest based on topsoil characteristics of study sites. To determine the response of soil characteristics to urbanization, general linear mixed models (LMMs) were applied. Relationships between species/functional richness and environmental variables (edaphic factors and urbanization index) were analyzed by LMMs. After fitting the full models for each dependent variable, we used the Akaike Information Criterion (AIC) to select the most parsimonious model. To determine whether soil properties and urbanization had significant effects on taxonomic and functional assemblage composition, permutational multivariate variance of analyses (PERMANOVAs, Jaccard and Bray–Curtis dissimilarity index, respectively; permutation = 9999) were applied. Results were visually displayed using non-metric multidimensional scaling (NMDS). Multivariate generalized linear models for presence-absence data (“manyglm” function, family = binomial) were used to evaluate individual species responses. For functional group responses, LMMs on CWM values for each attribute of a given trait/preference were carried out. These models complement PERMANOVAs by providing univariate responses to factors, in addition to overall multivariate responses. To consider and test the effects of the study sites’ size variability, the “area” variable was included in the models. The “side” variable (Buda, Pest) was considered as a random factor. To assess how taxonomic β diversity (turnover and nestedness) changed with urban intensity, the study sites of Buda and Pest were divided into three categories (UI1: Least urbanized; UI2: Moderately urbanized; UI3: Highly urbanized) based on urbanization index. For multiple comparisons of dissimilarity values among the three habitat types, nonparametric two-sided Tukey-type tests were applied using the “nparcomp” function.

## 3. Results

### 3.1. Taxonomic Diversity and Species Composition of Diplopoda Assemblages

**Figure 2:**
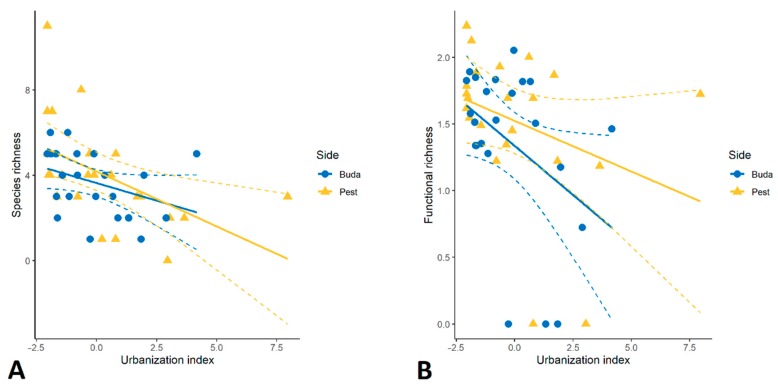
Relationship between species richness (**A**), functional richness (**B**) and urbanization on the two sides of Budapest. Dashed lines represent 95% confidence intervals.

A total of 24 species belonging to 8 families were identified in the 47 study sites of Budapest (Table 2). Total species richness was 14 and 20 on Buda and Pest sides, respectively. The average species number of millipedes (mean ± standard deviation (SD)) was 3.7 ± 1.5 per site in Buda compared to 4.1 ± 2.4 per site in Pest, with no significant difference between the two sides of the city. The most species-rich sampling sites contained 6 and 11 species in Buda and Pest, respectively (Tables S2–S3, Supplementary Materials). We found a new species to the fauna of the city, *Cylindroiulus caeruleocinctus* (Wood, 1864), which occurred only in one city park on the Pest side. The most frequent species were *Cylindroiulus boleti* (68.1%) and *Ophyiulus pilosus* (59.6%). The number of species was negatively influenced by the urbanization index (*t* = −3.5, *p* = 0.001; Figure 2A). 

**Figure 3:**
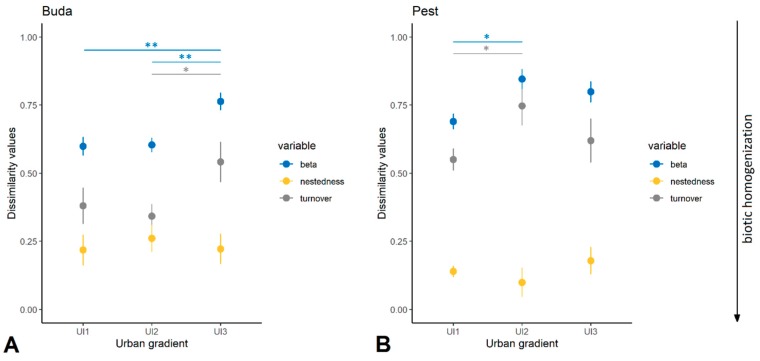
Multiple comparisons of dissimilarity values (β diversity, nestedness, and turnover) along an urban gradient in Buda (**A**) and Pest (**B**). Abbreviations: UI1, least urbanized; UI2, moderately urbanized; UI3, highly urbanized. Statistical significance is determined at ** *p* < 0.01 and * *p* < 0.05.

There were significant differences among urban habitat categories in dissimilarity values, with no consistent tendency in Buda or Pest. The level of taxonomic dissimilarity (taxonomic β diversity) was significantly higher among the most urbanized sites (UI3) than the least (UI1) and moderately urbanized (UI2) ones in Buda (*p* = 0.005 and *p* = 0.002, respectively). The contribution of species turnover to taxonomic dissimilarity among sites was highest in the UI3 category, differing significantly from UI2 (*p* = 0.044). The dissimilarity values in Pest showed a slightly different pattern. The moderately urbanized habitats (UI2) were characterized by the highest taxonomic β diversity and species turnover, and significantly differed from U1 ones (*p* = 0.013 and *p* = 0.025, respectively; Figure 3).

**Figure 4:**
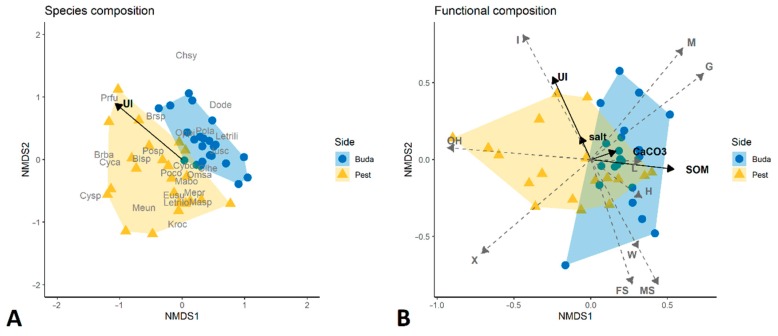
Non-metric multidimensional scaling (NMDS) analyses of taxonomic (**A**) and functional (**B**) composition of Diplopoda assemblages. For abbreviations of species names, see Table 2. The significant environmental variables are indicated by black arrows: UI, urbanization index; SOM, soil organic matter. The grey dashed arrows indicate the functional traits/ecological preferences: L, length; W, width; FS, forest specialist; G, generalist; H, hygrophilous; I, insensitive; M, mesophilic; MS, moderately sensitive; OH, open habitat; X, xerotolerant.

Significant differences in species composition of Diplopoda assemblages were experienced between the two sides of Budapest (*R*^2^ = 0.14, *p* < 0.001; Figure 4A). There was higher variability in taxonomic composition in Pest than the Buda side. NMDS analysis resulted in two clearly separated groups of sites. Many species occurred either only in Buda or in Pest (Table 2). *Ophyiulus pilosus* (*Dev* = 10.4, *p* = 0.021), *Megaphyllum unilineatum* (*Dev* = 18.0, *p* = 0.001), *Kryphyoiulus occultus* (*Dev* = 12.3, *p* = 0.008), and *Brachyiulus bagnalli* (*Dev* = 10.6, *p* = 0.021) were more common in Pest, while *Leptoiulus trilineatus* (*Dev* = 17.2, *p* = 0.001) was exclusively found in Buda. The degree of urbanization had significant effects on species composition (*R*^2^ = 0.08, *p* < 0.001; Figure 4A). For individual species responses, *Cylindroiulus boleti* (*Dev* = 29.53, *p* = 0.001), *Megaphyllum projectum* (*Dev* = 11.0, *p* = 0.039), and *Mastigona bosniensis* (*Dev* = 14.5, *p* = 0.006) showed negative relationship with urban disturbance.

### 3.2. Traits Analysis and Functional Diversity

The list of functional traits/ecological preferences for the different species is presented in Table S1. Both species richness and functional richness (expressed as FDis) decreased with the urbanization index (*t* = −2.4, *p* = 0.02; Figure 2B). Diplopoda assemblages were functionally different across the sides of Budapest (*R*^2^ = 0.19, *p* < 0.001; Figure 4B). Xerotolerant species and species preferring open habitats occurred mainly in sites in Pest (*t* = 2.6, *p* = 0.01 and *t* = 2.2, *p* = 0.03, respectively), while species with higher humidity preference (“mesophilic” category in this study) were more frequent on the Buda side (*t* = 3.4, *p* < 0.001). Urbanization had significant effects on the functional composition of millipedes (*R*^2^ = 0.19, *p* < 0.001; Figure 4B).

Regarding morphological traits, species bigger in size (longer and wider) prevailed in less urbanized habitats (length: *t* = −2.9, *p* = 0.005; width: *t* = −5.2, *p* < 0.001). Insensitive- and open habitat-preferring species were dominant in more urbanized sites (*t* = 4.4, *p* < 0.001 and *t* = 2.6, *p* = 0.01, respectively). Forest specialists and species moderately sensitive to urban intensity were less frequent or absent in disturbed habitats (*t* = −2.7, *p* = 0.01 and *t* = −4.4, *p* < 0.001, respectively). 

### 3.3. Diplopoda Assemblages and Soil Properties

**Figure 5:**
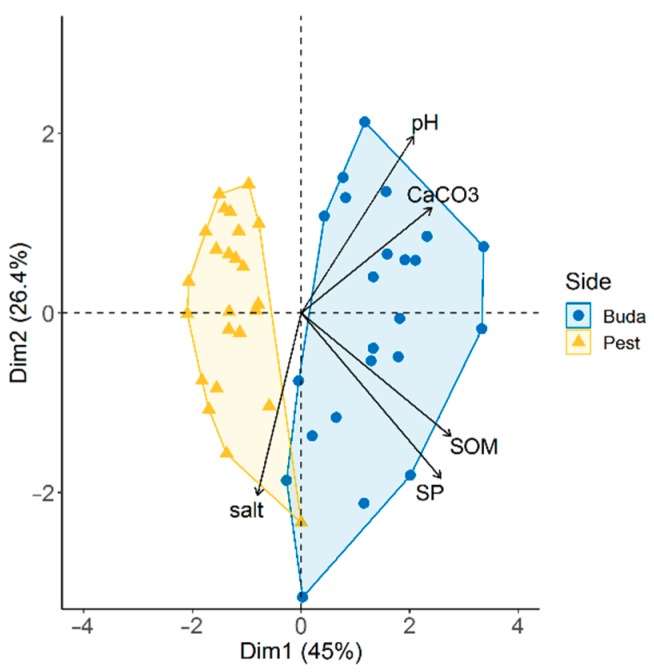
Principal component analysis (PCA) biplot of the sample sites according to the studied edaphic factors. Abbreviations: SOM, soil organic matter; SP, saturation percentage.

Topsoil characteristics of habitats showed a significant difference between the two sides of Budapest (Tables S4 and S5, Supplementary Materials). The PCA biplot resulted in two distinct, non-overlapping groups of study sites (Figure 5). The first two principal components explained 71.4% of the total variance of the dataset. Sites located in Buda were characterized by soils with higher pH, saturation percentage (SP), and CaCO_3_ and SOM content. Soil pH and SP were significantly affected by urbanization. Soils of more urbanized habitats had higher pH (*t* = 2.0, *p* = 0.050) and lower soil plasticity (*t* = −2.7, *p* = 0.007) compared to less disturbed sites.

We found no significant effects of the studied edaphic factors on species composition of millipedes. However, the functional composition of Diplopoda assemblages was significantly influenced by CaCO_3_, SOM, and salt content of soils (Figure 4B, Table 3). Community weighted mean (CWM) values for several ecological preferences were influenced by the studied soil properties (Table 3). SOM content had positive effects on xerotolerant and moderately sensitive species, and negative effects on insensitive and mesophilic species. CaCO_3_ content of soils positively affected forest specialists. We found a positive correlation between the occurrence of mesophilic species and the total soluble salt content of soils.

## 4. Discussion

### 4.1. Diplopoda Fauna of Budapest

The 24 millipede species found in our survey represent 23% of the Hungarian millipede fauna [40]. Most of the recorded species are widespread in Europe [41,42]. Millipedes from urban and other anthropogenic habitats were previously investigated in several European cities [43,44,45,46,47,48,49,50,51,52,53]. Discussing some of these data, Vilisics et al. [53] stated that urban areas can be characterized by 14–26 species on average. 

Korsós [47] and later Korsós et al. [48] provided data on the Diplopoda fauna of Budapest, including different types of man-made habitats, e.g., glasshouses, city gardens, secondary disturbed woods, cemeteries, large park forests, and floodplain forests of the Danube. These studies found 27 and 26 species, respectively, which was similar to our results. However, typical glasshouse millipedes (e.g., *Choneiulus palmatus, Oxidus gracilis, Cylindroiulus truncorum*) were missing from our species list due to our study focusing only on a subset of the diverse urban habitat types. The most frequent species, *Cylindroiulus boleti* and *Ophyiulus pilosus*, are sylvicol, although *O. pilosus* has been found in a wide variety of habitats such as gardens, parks, and meadows [42]. Our survey is the first record of *Cylindroiulus caeruleocinctus* in Budapest. This is a strongly synanthropic species, predominantly occurring in gardens, parks, cemeteries, orchards, waste places, and cultivated ground [28]. 

The present study is the first to compare the millipedes of the two sides of Budapest, both faunistically and ecologically. Our results show that Buda and Pest have significantly different Diplopoda fauna. Xerotolerant species typical of open land (*B. bagnalli*, *K. occultus*, *M. unilineatum*) were constant elements of habitats in Pest, mainly characterized by flat landscapes. By contrast, a mesophilic species, *L. trilineatus* [54]. was exclusively found on the Buda side, which may be explained by a more humid macroclimate associated with the region of the Buda Hills [22]. The large difference in community composition is due to both sides of the city having respective regional species pools. 

### 4.2. Urban Effects on Taxonomic/Functional Diversity and Composition of Millipedes

Taxonomic diversity of Diplopoda has been previously investigated in urban contexts, but to our knowledge, this is the first study that explicitly explores the urban effects on functional richness. We found that urbanization has a negative effect on species and functional richness of millipede assemblages on both sides of Budapest. Mwabvu [55], studying spirostreptid millipedes, also reported that species richness decreased with increasing urban disturbance. In a rural–urban gradient study (Debrecen, Hungary), Bogyó et al. [43] found the highest millipede abundance, species richness, and diversity in the suburban area. Higher functional diversity of millipedes was observed in less urbanized habitats that are rich in decaying wood and leaf litter, as reported by Nagy et al. [56] for woodlice. Similarly, Fournier et al. [57] found a negative functional response of millipedes to disturbance in a restored floodplain. Urban green area management (e.g., litter removal), together with the limited dispersal ability of millipedes [19], hindered recolonization [58,59]. 

Our aim was to also test whether—and if so, how—urbanization has altered processes of community assembly, leading to biotic homogenization. The homogenization of millipede assemblages has been inferred from the reductions in between-site dissimilarity in terms of species composition (β diversity) along an urban gradient. Contrary to our expectations, β diversity (and species turnover) seemed to increase with increasing urban intensity. Homogenization of urban communities has been previously observed in several taxa (e.g., plants [5], microbes [6], insect orders [7,8], and birds [9]). However, the relationship between urbanization and similarity is complex. For example, Jokimäki and Kaisanlahti-Jokimäki [60] found lower avifaunal similarity among more urbanized (town centers) compared to less urbanized habitats (apartment blocks and single-family houses) in five European towns. Similarly to our results, Magura et al. [61] did not experience the homogenization effect of urbanization on ground beetles. On the one hand, spatial (e.g., dispersal limitation), environmental (e.g., habitat filtering) and/or biological (e.g., limiting similarity) factors may contribute to such enhancement of β diversity [62]. On the other hand, biotic homogenization is a time-dependent process and it may take longer for the rearrangement of communities [63]. An additional explanation for our results could be that certain species (“matrix species”) can easily colonize microhabitats of altered forest fragments in urban areas, and such unpredictable colonization success may lead to heterogeneous and differing assemblages [61].

Regarding individual species responses, three forest species that are very common in Central European woodlands [41,42], proved to be sensitive to urbanization: *C. boleti, M. projectum*, and *M. bosniensis*. These species preferred suburban and rural areas in the urban study of Bogyó et al. [43]. Habitat loss and fragmentation resulted from urban sprawling lead to a drastic decline of forest specialists [64], as we have shown in the present study. Our traits-based analysis also confirmed that forest species are more sensitive to disturbance. By contrast, certain millipedes—mainly belonging to open land and generalist species—showed different habitat preferences, and their occurrence increased with urban intensity. These synanthropic species have adapted to disturbed and often man-made habitats [65]. Our results are consistent with previous studies on other soil arthropod taxa (e.g., spiders [66], beetles [61], woodlice [67,68], and millipedes [43]) in that the ratio of forest specialist to synanthropic species decreases with urbanization. Moreover, we observed a negative correlation between higher urban disturbance and smaller body size (length and width based on literature data) of millipedes. Similar results were published by Bogyó et al. [43] and Magura et al. [69], who found significantly smaller individuals of millipedes and ground beetles in urban areas compared to rural and suburban ones, respectively. This phenomenon may be related to the deterioration of soil habitat quality. Urban soils are exposed to a variety of direct (e.g., sealing, trampling, chemical contamination) and indirect (e.g., urban heat island effect, anthropogenic disturbance) adverse effects [15]. In our study, soil pH and texture were significantly influenced by urban intensity. Several previous studies have shown that urban soils, especially those disturbed and/or heavily managed, typically have higher pH, which has been associated with materials used in urban infrastructure that are high in calcium, such as concrete [70,71]. Anthropogenic activities often result in soil degradation, as shown by the lower saturation percentage values in more disturbed habitats, indicating lost or strongly modified soil structure [70,72]. These habitat modifications could significantly reduce the survival and fitness of millipedes [19]. The smaller body size in more urbanized sites may be a result of lower food quality [73], higher concentration of contaminants (e.g., heavy metals released by fuel combustion emissions), and/or lower soil moisture [19]. Physical soil disturbance, sparse vegetation (and litter layer), and higher urban temperatures are known to favor rapid water loss/desiccation of the topsoil. The humidity level is an important filter for Diplopoda [64], especially in disturbed soil habitats. Their exoskeleton is permeable to water; therefore, risk of desiccation restricts their occurrence to habitats with higher humidity and suitable shelter sites, such as woody debris and leaf litter [19]. Urban environmental changes can act as a filter, removing all the species lacking specific combinations of traits, as described by Keddy [74]. 

### 4.3. Diplopoda Assemblages and Soil Properties

Relatively large differences in edaphic factors were observed between the two sides of the city, which can partly explain the compositional dissimilarities. The functional composition of assemblages was significantly influenced by CaCO_3_, SOM, and total soluble salt content of soils. As millipedes require calcium sources to develop their calcified exoskeleton [19], calcium availability affects their presence. In this study, forest specialists showed positive response to soil CaCO_3_ content. For example, typical forest species, *Glomeris hexasticha* and *Chordeuma sylvestre*, have been shown to be calciphilic [25,75]. Some ecological preferences (humidity and disturbance tolerance) were significantly correlated to SOM content. In general, millipedes, as detritus feeders, are indirectly influenced by SOM content, rather than directly [16]. The presence and type of humus are known to have crucial importance for millipedes [76]. Organic matter levels affect fertility, structure, buffer, and water-holding capacity of soils, thus have an essential role in defining soil habitat quality [16]. The concentration of total soluble salt in soils also proved to be an important edaphic factor in functional community assembly. This may be related to the osmotic effect of salts, which significantly determine the water balance of soil-dwelling organisms [77]. 

## 5. Conclusions

Our study showed that urban soil disturbance has detrimental effects on species and functional richness of Diplopoda assemblages on both sides of Budapest. Urbanization was the most important driver of taxonomic and functional community assembly. Species had different responses to anthropogenic habitat modification, depending on their ecological needs and tolerance. Although urbanization seemingly did not homogenize Diplopoda assemblages in Budapest, its generality should be tested in more locations or cities. The decline in taxonomic and functional diversity of millipedes could result in strong alteration in soil organic matter dynamics due to the vital functional importance of millipedes in urban ecosystems across the ranges of nutrient cycling and organic matter decomposition [16]. Urban green spaces are usually heavily managed and litter is removed, which results in increasing urban disturbance for litter-dwelling invertebrates. It would benefit them to provide appropriate supplies of detritus and shelter sites, as this would promote species richness, abundance, and function. We therefore recommend management of natural habitat remnants within cities be “invertebrate friendly”.

## Figures and Tables

**Table 1:** Selected traits and ecological preferences of Diplopoda species.

	Trait	Type	Units/levels
Morphological traits	length	quantitative	mm
	width	quantitative	mm
Ecological preferences	habitat affinity	ordinal	1 open habitat, 2 generalist, 3 forest specialist
	humidity preference	ordinal	1 xerotolerant, 2 mesophilic, 3 hygrophilous
	disturbance sensitivity	ordinal	1 insensitive, 2 moderately sensitive, 3 sensitive

**Table 2:** Species list of millipedes with their frequency of occurrence in Buda and Pest. Abbreviations of species names used in Figure 4A.

Family	Species	Abbreviations	Frequency (%)	
			Buda	Pest
Blaniulidae	*Blaniulidae* sp.	Blsp	4.3	12.5
	*Proteroiulus fuscus* (Am Stein, 1857)	Prfu	0	4.2
Chordeumatidae	*Chordeuma sylvestre* C. L. Koch, 1847	Chsy	4.3	0
Dorypetalidae	*Dorypetalum degenerans* (Latzel, 1884)	Dode	13	0
Glomeridae	*Glomeris hexasticha* Brandt, 1833	Glhe	13.0	8.3
Julidae	*Brachyiulus bagnalli* Brölemann, 1924	Brba	0	29.2
	*Cylindroiulus boleti* (C. L. Koch, 1847)	Cybo	78.3	58.3
	*Cylindroiulus caeruleocinctus* (Wood, 1864)	Cyca	0	4.2
	*Cylindroiulus* sp.	Cysp	0	8.3
	*Julus scandinavius* (Latzel, 1884)	Jusc	8.7	0
	*Kryphioiulus occultus* (Koch, C. L., 1847)	Kroc	0	33.3
	*Leptoiulus trilineatus* (Koch, C. L., 1847)	Letrili	43.5	0
	*Leptoiulus trilobatus* Verhoeff, 1894	Letrilo	0	4.2
	*Megaphyllum projectum* Verhoeff, 1894	Mepr	8.7	20.8
	*Megaphyllum unilineatum* (Koch, 1838)	Meun	4.3	58.3
	*Ommatoiulus sabulosus* (Linnaeus, 1758)	Omsa	39.1	25.0
	*Ophyiulus pilosus* (Newport, 1843)	Oppi	82.6	37.5
Mastigophorophyllidae	*Mastigona bosniensis*(Verhoeff, 1897)	Mabo	21.7	25.0
	*Mastigona* sp.	Masp	0	4.2
Polydesmidae	*Brachydesmus* sp.	Brsp	17.4	20.8
	*Eubrachydesmus superus* (Latzel, 1884)	Eusu	0	12.5
	*Polydesmus complanatus* (Linnaeus, 1761)	Poco	0	20.8
	*Polydesmus* sp.	Posp	0	16.7
Polyxenidae	*Polyxenus lagurus* (Linnaeus, 1758)	Pola	30.4	4.2

**Table 3:** Results of the general linear mixed models showing significant relationships between functional trait levels and edaphic factors. SOM: soil organic matter.

Traits/Ecological Preferences	Levels	Edaphic Factors	*t*	*p*
Habitat affinity	forest specialist	CaCO_3_	2.7	0.01
Humidity preference	xerotolerant	SOM	2.0	0.05
	mesophilic	SOM	−2.7	0.01
		salt	2.6	0.02
Disturbance sensitivity	insensitive	SOM	−3.8	<0.001
	moderately sensitive	SOM	3.8	<0.001

## Supplementary Materials

The following is available online at https://www.mdpi.com/2075-4450/11/1/25/s1: Table S1, Trait values for each species, used in the analysis of the traits/ecological preferences. Table S2, Species occurrence of study sites on the Buda side of the Budapest metropolitan area. × indicates presence. Table S3, Species occurrence of study sites on the Pest side of the Budapest metropolitan area. × indicates presence. Table S4, Geographical location and habitat characterization (urbanization intensity and topsoil characteristics) of study sites on the Buda side of the Budapest metropolitan. Table S5, Geographical location and habitat characterization (urbanization intensity and topsoil characteristics) of study sites on the Pest side of the Budapest metropolitan.
