# Supplementary material for: Taxonomic and Functional Response of Millipedes (Diplopoda) to Urban Soil Disturbance in a Metropolitan Area
Source: Insects. 2019 Dec 29;11(1):25. doi: 10.3390/insects11010025 (PMC7022796; doi:10.3390/insects11010025)
Supplement: Supplementary file 1 [file insects-11-00025-s001.zip › insects-643986-SUPP/Table_S1.docx]

**Table S1**. Trait values for each species, used in the analysis of the traits/ecological preferences.

| **Family** | **Species** | **Length** | **Width** | **Habitat affinity** | **Humidity preference** | **Disturbance sensitivity** | **Source** |
| --- | --- | --- | --- | --- | --- | --- | --- |
| Blaniulidae | *Proteroiulus fuscus*  (Am Stein, 1857) | 11.2 | 0.7 | 3 | 3 | 1 | Blower 1985, Bogyó et al. 2012, Korsós 1990, 1992, 1994, Peitsalmi 1974, Schubart 1934, Stojalowska 1961, Thiele 1968, Tracz 1984, Voigtländer 2011 |
| Chordeumatidae | *Chordeuma sylvestre*  C. L. Koch, 1847 | 14.5 | 1.4 | 3 | 3 | 2 | Blower 1985, Dunger & Steinmetzger 1981, Schubart 1934, Thiele 1968 |
| Dorypetalidae | *Dorypetalum degenerans* (Latzel, 1884) | 17 | 1.1 | 2 | 2 | 1 | Daday 1889, Korsós 1992, 1994 |
| Glomeridae | *Glomeris hexasticha*  Brandt, 1833 | 11.5 | 4.8 | 3 | 3 | 2 | Bogyó et al. 2012, Dunger & Steinmetzger 1981, Gebhardt 1966, Ilosvay 1985, Jedryczkowski 1979, Korsós 1990, 1994, Korsós & Dányi 2002, Lazányi & Korsós 2009, Loksa 1981, Schubart 1934, Stojalowska 1961, Thiele 1959, 1968 |
| Julidae | *Brachyiulus bagnalli* Brölemann, 1924 | 10.1 | 0.8 | 1 | 1 | 1 | Bogyó et al. 2012, Korsós 1992, Korsós & Dányi 2002 |
|  | *Cylindroiulus boleti*  (C. L. Koch, 1847) | 26 | 2.2 | 3 | 1 | 2 | Gebhardt 1966, Korsós 1992, 1994, Loksa 1956, 1981, Schubart 1934, Verhoeff, 1934 |
|  | *Cylindroiulus caeruleocinctus*  (Wood, 1864) | 24.7 | 2.2 | 1 | 3 | 1 | Blower 1985, Bogyó & Korsós 2010, Haacker 1968 |
|  | *Julus scandinavius*  (Latzel, 1884) | 25.6 | 2 | 2 | 3 | 2 | Blower 1985, Dunger & Steinmetzger 1981, Sallai 1992, Schubart 1934, Steinmetzger 1982, Thiele 1968 |
|  | *Kryphioiulus occultus*  (Koch, C. L., 1847) | 11 | 0.8 | 1 | 1 | 1 | Bogyó et al. 2012, Korsós, 1991, Lazányi & Korsós 2009, Schubart 1934, Stojalowska 1961, Verhoeff, 1934, Voigtländer 1987 |
|  | *Leptoiulus trilineatus*  (Koch, C. L., 1847) | 32.5 | 2.1 | 2 | 2 | 2 | Bachvarova et al. 2018, Schubart 1934 |
|  | *Leptoiulus trilobatus* Verhoeff, 1894 | 30.5 | 2.3 | 3 | 3 | 2 | Jedryczkowski 1979, Korsós & Lazányi 2008, Lazányi & Korsós 2009, Schubart 1934, Tuf & Tufová 2008, Verhoeff, 1934 |
|  | *Megaphyllum projectum* Verhoeff, 1894 | 29.3 | 2.8 | 3 | 1 | 2 | Bogyó et al. 2012, Gebhardt 1966, Haacker 1967, Jedryczkowski 1979, Korsós 1990, 1992, 1998, Korsós & Dányi 2002, Korsós & Lazányi 2008, Lazányi & Korsós 2009, Sallai 1992, Schubart 1934, Stojalowska 1961, Verhoeff, 1934 |
|  | *Megaphyllum unilineatum*  (Koch, 1838) | 27.6 | 2.4 | 1 | 1 | 1 | Bogyó et al. 2012, Dunger & Steinmetzger 1981, Haacker 1967, Korsós 1987, 1991, 1992, 1998, Loksa 1981, 1983, Sallai 1992, Schubart 1934, Stojalowska 1961, Verhoeff, 1934 |
|  | *Ommatoiulus sabulosus* (Linnaeus, 1758) | 29.8 | 2.7 | 2 | 1 | 1 | Blower 1985, Bogyó et al. 2012, Dunger & Steinmetzger 1981, Gebhardt 1966, Geoffroy 1981, Haacker 1967, Halkka 1958, Jedryczkowski 1979, Loksa 1981, Schubart 1934, Stojalowska 1961, Thiele 1968, Verhoeff, 1934 |
|  | *Ophyiulus pilosus* (Newport, 1843) | 27.2 | 1.8 | 2 | 2 | 1 | Blower 1985, Korsós 1992, Korsós & Dányi 2002, Korsós & Lazányi 2008, Lazányi & Korsós 2009, Schubart 1934, Stojalowska 1961 |
| Mastigophorophyllidae | *Mastigona bosniensis* (Verhoeff, 1897) | 12.1 | 1.3 | 2 | 1 | 2 | Bogyó et al. 2012, Dunger & Steinmetzger 1981, Loksa 1981, Sallai 1992, Schubart 1934, Stojalowska 1961, Verhoeff, 1934 |
| Polydesmidae | *Eubrachydesmus superus*  (Latzel, 1884) | 8.7 | 1 | 2 | 2 | 1 | Blower 1985, Jedryczkowski 1979, Schubart 1934, Stojalowska 1961, Thiele 1968, Verhoeff, 1934 |
|  | *Polydesmus complanatus*  (Linnaeus, 1761) | 22.2 | 3.3 | 2 | 3 | 1 | Angyal & Korsós 2013, Bogyó et al. 2012, Gebhardt 1966, Jedryczkowski 1979, Korsós 1987, 1990, 1994, Korsós & Dányi 2002, Korsós & Lazányi 2008, Loksa 1981, Margó 1879, Sallai 1992, Schubart 1934, Seres 2000, Stojalowska 1961 |
| Polyxenidae | *Polyxenus lagurus* (Linnaeus, 1758) | 2.7 | 0.7 | 2 | 2 | 1 | Blower 1985, Bogyó et al. 2012, Gebhardt 1966, Geoffroy 1981, Korsós 1990, 1992, 1994, 1998, Loksa 1981, Schubart 1934, Stojalowska 1961, Thiele 1968, Verhoeff, 1934 |

**References to Table S1.** Publications, not referenced in the main text, are listed below.

Angyal, D.; Korsós, Z. Millipedes (Diplopoda) of twelve caves in Western Mecsek, Southwest Hungary. *Opusc. Zool. Budapest* **2013**, 44(2), 99–106.

Blower, J.G. *Millipedes*. Synopses of the British Fauna (New Series) 35, E.J. Brill: London, UK, 1985; 242 pp.

Bogyó, D.; Korsós, Z. *Cylindroiulus caeruleocinctus* (Wood, 1864), new to the fauna of Hungary, and its current European distribution (Diplopoda: Julida). *Schubartiana*, **2010**, 4, 9–14.

Bogyó, D.; Korsós, Z.; Lazányi, E.; Hegyessy G. Millipedes (Diplopoda) from the Zemplén Mountains, Northeast Hungary, with two julid species new to the Hungarian fauna. *Opusc. Zool. Budapest* **2012**, 43(2), 131–145.

Daday, J. A magyarországi Myriapodák magánrajza. Királyi Magyar Természettudományi Társulat: Budapest, Hungary, 1889.

Dunger, W.; Steinmetzger, K. Ökologische Untersuchungen an Diplopoden einer Rasen-Wald-Catena im Thüringer Kalkgebeit (Ecological investigations on Diplopoda of a grassland-wood-catena in a Limestone Area in Thuringia (GDR)). *Zool. Jb. Syst.* **1981**, 108, 519–553.

Gebhardt, A. A Mecsek hegység állatvilága II. Ízeltlábúak (Arthropoda): Rákok (Crustacea), Soklábúak (Myriopoda). *A Janus Pannonius Múzeum Évkönyve – Természettudományok* **1966**, 11, 7–15.

Geoffroy, J.-J. Étude d’un écosystème forestier mixte V. – Traits généraux du peuplement de Diplopodes édaphiques. *Rev. Écol. Biol. Sol* **1981**, 18(3), 357–372.

Haacker, U. Deskriptive, experimentelle und vergleichende Untersuchungen zur Autökologie rhein-mainischer Diplopoden (Descriptive, experimental and comparative investigations on the ecology of Diplopoda of the Rhein-Main-Region). *Oecologia (Berl.)* **1968**, 1, 87–129.

Halkka, R. Life history of *Schizophyllum sabulosum* (L.) (Diplopoda, Iulidae). *Ann. Zool. Soc. ‘Vanamo’* **1958**, 19(4): 1–71.

Ilosvay, G. A zirci arborétum Isopoda, Diplopoda és Chilopoda faunájáról. *A Bakony Term. tud. Kutatásának Eredményei* **1985**, 16, 43–50.

Jedryczkowski, W. Krocionogi (Diplopoda) Bieszczadów. *Fragmenta Faunistica* **1979**, 25(6), 77–93.

Korsós, Z. Abundance and seasonal activity of millipedes in a dolomitic grassland community (Diplopoda). *Annls hist.-nat. Mus. natn. hung.* **1991**, 83, 239–244.

Korsós, Z. Checklist, preliminary distribution maps, and bibliography of millipedes in Hungary (Diplopoda). *Misc. Zool. Hung.* **1994**, 9, 29–82.

Korsós, Z. A Dráva mente ikerszelvényes (Diplopoda) faunája [Millipede (Diplopoda) fauna of the Hungarian Dráva Region]. *Dunántúli Dolg. Term. tud. Sorozat* **1998**, 9, 81–96.

Korsós, Z.; Dányi, L. Millipedes (Diplopoda) and centipedes (Chilopoda) of the Fertő-Hanság National Park, Hungary. In *Fauna of the Fertő-Hanság National Park*; Mahunka, S., Ed.; Hungarian National History Museum: Budapest, Hungary, 2002; pp. 183–190.

Korsós, Z.; Lazányi, E. Millipedes (Diplopoda) of Maramures (Romania). *Stud. Univ. Vasile Goldis Arad Ser. Stiint. Vietii* **2008**, 18, 199–209.

Lazányi, E.; Korsós, Z. Millipedes (Diplopoda) of the Aggtelek National Park, Northeast Hungary. *Opusc. Zool. Budapest* **2009**, 40(1): 35–46.

Loksa, I. The Diplopod and Chilopod faunas of the environs of Lake Velence. *Term. tud. Múzeum Évkönyve* **1956**, 25, 385–390.

Loksa, I. A barcsi borókás ikerszelvényes (Diplopoda) és százlábú (Chilopoda) faunája. *Dunántúli Dolg. Term. tud. Sorozat* **1981**, 2, 45–52.

Margó, T. *Budapest és környéke állattani tekintetben (Budapest and surroundings in zoological respect).* Magyar Királyi Egyetemi Nyomda: Budapest, Hungary, 1879; pp. 116–117 (in Hungarian).

Peitsalmi, M. Vertical orientation and aggregation of *Proteroiulus fuscus* (Am Stein) (Diplopoda, Blaniulidae). *Symp. Zool. Soc. Lond.* **1974**, 32, 471–483.

Sallai, Á. On the soil-inhabiting macrofauna of Nagy-Szénás, with special reference to the isopods, diplopods and chilopods. *Opusc. Zool. Budapest* **1992**, 25, 95–102.

Seres, A. Arthropod fauna of two inflow caves in the Bükk Mts. In *Fauna jaskýň (Cavc Fauna)*; Mock, A., Kovác, L., Fulín, M., Eds.; State Nature Conservancy of the Slovak Republic Slovak Caves Administration: Košice, Slovakia, 2000, pp. 151–156.

Steinmetzger, K. Die Diplopoden des Waldgebietes Hakel im nordöstlichen Harzvorland der DDR. *Hercynia N.F. (Leipzig)* **1982**, 19(2), 197–205.

Stojałowska W. 1961, *Krocionogi (Diplopoda) Polski*, Państwowe Wydawnictwo Naukowe: Warsaw, Poland, 216 pp.

Thiele, H.U. Die Diplopoden des Rheinlandes. *Decheniana* **1968**, 120(1-2), 343–366.

Thiele, H.U. Experimentelle Untersuchungen über die Abhängkikeit bodenbewohnender Tierarten vom Kalkgehalt des Standortes (mit besonderer Berücksichtigung der Diplopoden). *Z. Ang. Ent.* **1959**, 44, 1–21.

Tracz, H. Studies on the ecology of *Proteroiulus fuscus* (Am Stein, 1857) (Diplopoda, Blaniulidae). *Acta Zool. Cracov.* **1984**, 27(21), 519–576.

Tuf, I.H.; Tufová, J. Proposal of ecological classification of centipede, millipede and terrestrial isopod faunas for evaluation of habitat quality in Czech Republic. *Cas. Slez. Muz. Opava (A)* **2008**, 57, 37–44.

Verhoeff, K.W. Oberklasse Progoneata (Diplopoda, Symphyla, Pauropoda); Oberklasse Opisthogoneata (Chilopoda). In *Die Tierwelt Mitteleuropas 2 (3)*; Brohmer, P., Ehrmann, P., Ulmer, G., Eds.; Verlag Von Quelle & Meyer: Leipzig, Germany, 1934; 120 pp.

Voigtländer, K. Untersuchungen zur Bionomie von *Enantiulus nanus* (Latzel, 1884) und *Allajulus occultus* C.L. Koch, 1847 (Diplopoda, Julidae). *Abh. Ber. Naturkundemus. Görlitz* **1987**, 60(10), 1–116.

Voigtländer, K. Preferences of common central European millipedes for different biotope types (Myriapoda, Diplopoda) in Saxony-Anhalt (Germany). *Int. J. Myriapod.* **2011**, 6, 61–83. http://dx.doi.org/10.3897/ijm.6.2172
