# Supplementary material for: Taxonomic and Functional Response of Millipedes (Diplopoda) to Urban Soil Disturbance in a Metropolitan Area
Source: Insects. 2019 Dec 29;11(1):25. doi: 10.3390/insects11010025 (PMC7022796; doi:10.3390/insects11010025)
Supplement: Supplementary file 1 [file insects-11-00025-s001.zip › insects-643986-SUPP/Table_S2.docx]

**Table S2.** Species occurrence of study sites on the Buda side of the Budapest metropolitan area. × indicates presence.

| **Species** | **1** | **2** | **3** | **4** | **5** | **6** | **7** | **8** | **9** | **10** | **11** | **12** | **13** | **14** | **15** | **16** | **17** | **18** | **19** | **20** | **21** | **22** | **23** |
| --- | --- | --- | --- | --- | --- | --- | --- | --- | --- | --- | --- | --- | --- | --- | --- | --- | --- | --- | --- | --- | --- | --- | --- |
| Blaniulidae |  |  |  |  |  |  |  |  |  |  |  |  |  |  |  |  |  |  |  |  |  |  |  |
| *Blaniulidae* *sp.* |  |  |  |  |  |  |  |  |  |  |  |  |  |  |  |  |  |  |  |  | **×** |  |  |
| *Proteroiulus fuscus* |  |  |  |  |  |  |  |  |  |  |  |  |  |  |  |  |  |  |  |  |  |  |  |
| Chordeumatidae |  |  |  |  |  |  |  |  |  |  |  |  |  |  |  |  |  |  |  |  |  |  |  |
| *Chordeuma sylvestre* |  |  |  | **×** |  |  |  |  |  |  |  |  |  |  |  |  |  |  |  |  |  |  |  |
| Dorypetalidae |  |  |  |  |  |  |  |  |  |  |  |  |  |  |  |  |  |  |  |  |  |  |  |
| *Dorypetalum degenerans* |  |  |  | **×** |  |  |  |  |  |  |  |  |  |  |  |  | **×** |  |  | **×** |  |  |  |
| Glomeridae |  |  |  |  |  |  |  |  |  |  |  |  |  |  |  |  |  |  |  |  |  |  |  |
| *Glomeris hexasticha* |  | **×** |  |  |  | **×** |  |  |  |  |  |  |  |  |  |  |  |  |  |  |  |  | **×** |
| Julidae |  |  |  |  |  |  |  |  |  |  |  |  |  |  |  |  |  |  |  |  |  |  |  |
| *Brachyiulus bagnalli* |  |  |  |  |  |  |  |  |  |  |  |  |  |  |  |  |  |  |  |  |  |  |  |
| *Cylindroiulus boleti* | **×** | **×** | **×** |  | **×** | **×** |  | **×** |  | **×** | **×** | **×** | **×** | **×** | **×** | **×** | **×** | **×** | **×** |  |  | **×** | **×** |
| *Cylindroiulus caeruleocinctus* |  |  |  |  |  |  |  |  |  |  |  |  |  |  |  |  |  |  |  |  |  |  |  |
| *Cylindroiulus sp.* |  |  |  |  |  |  |  |  |  |  |  |  |  |  |  |  |  |  |  |  |  |  |  |
| *Julus scandinavius* |  |  | **×** |  |  |  |  |  |  |  |  |  |  |  |  |  |  |  | **×** |  |  |  |  |
| *Kryphioiulus occultus* |  |  |  |  |  |  |  |  |  |  |  |  |  |  |  |  |  |  |  |  |  |  |  |
| *Leptoiulus trilineatus* | **×** |  |  |  | **×** | **×** |  | **×** |  |  | **×** |  |  | **×** | **×** |  |  |  | **×** | **×** |  |  | **×** |
| *Leptoiulus trilobatus* |  |  |  |  |  |  |  |  |  |  |  |  |  |  |  |  |  |  |  |  |  |  |  |
| *Megaphyllum projectum* |  |  | **×** |  |  |  |  |  |  |  |  |  | **×** |  |  |  |  |  |  |  |  |  |  |
| *Megaphyllum unilineatum* |  |  |  |  |  |  |  |  |  |  | **×** |  |  |  |  |  |  |  |  |  |  |  |  |
| *Ommatoiulus sabulosus* | **×** |  |  |  | **×** | **×** | **×** | **×** |  |  |  | **×** | **×** | **×** |  |  |  |  | **×** |  |  |  |  |
| *Ophyiulus pilosus* | **×** | **×** | **×** | **×** | **×** | **×** | **×** | **×** | **×** | **×** | **×** | **×** | **×** |  | **×** | **×** |  | **×** | **×** | **×** | **×** |  |  |
| Mastigophorophyllidae |  |  |  |  |  |  |  |  |  |  |  |  |  |  |  |  |  |  |  |  |  |  |  |
| *Mastigona bosniensis* | **×** |  | **×** |  |  | **×** |  |  |  |  |  | **×** |  | **×** |  |  |  |  |  |  |  |  |  |
| *Mastigona sp.* |  |  |  |  |  |  |  |  |  |  |  |  |  |  |  |  |  |  |  |  |  |  |  |
| Polydesmidae |  |  |  |  |  |  |  |  |  |  |  |  |  |  |  |  |  |  |  |  |  |  |  |
| *Brachydesmus sp.* |  |  |  | **×** |  |  |  | **×** |  |  |  |  |  |  |  | **×** |  |  |  | **×** |  |  |  |
| *Eubrachydesmus superus* |  |  |  |  |  |  |  |  |  |  |  |  |  |  |  |  |  |  |  |  |  |  |  |
| *Polydesmus complanatus* |  |  |  |  |  |  |  |  |  |  |  |  |  |  |  |  |  |  |  |  |  |  |  |
| *Polydesmus sp.* |  |  |  |  |  |  |  |  |  |  |  |  |  |  |  |  |  |  |  |  |  |  |  |
| Polyxenidae |  |  |  |  |  |  |  |  |  |  |  |  |  |  |  |  |  |  |  |  |  |  |  |
| *Polyxenus lagurus* |  |  |  | **×** | **×** |  |  |  |  |  | **×** |  |  | **×** |  | **×** |  | **×** | **×** |  |  |  |  |
