# Supplementary material for: Taxonomic and Functional Response of Millipedes (Diplopoda) to Urban Soil Disturbance in a Metropolitan Area
Source: Insects. 2019 Dec 29;11(1):25. doi: 10.3390/insects11010025 (PMC7022796; doi:10.3390/insects11010025)
Supplement: Supplementary file 1 [file insects-11-00025-s001.zip › insects-643986-SUPP/Table_S4.docx]

**Table S4.** Geographical location and habitat characterization (urbanization intensity and topsoil characteristics) of study sites on the Buda side of the Budapest metropolitan area.

| **Site** | **Coordinates** | **Area (km^2^)** | **UI^1^** | **Soil properties** | | | | |  |
| --- | --- | --- | --- | --- | --- | --- | --- | --- | --- |
|  |  |  |  | **pH** | **SP^2^ (%)** | **Salt (m/m %)** | **CaCO_3_ (m/m %)** | **SOM^3^ (m/m %)** | |
| 1 | 47° 34' 25" N; 18° 56' 23" E | 1.326 | -1.7 | 6.2 | 64.9 | 0.01 | 0.0 | 7.1 | |
| 2 | 47° 33' 49" N; 18° 59' 57" E | 0.220 | -0.0 | 7.5 | 47.3 | 0.01 | 11.0 | 4.8 | |
| 3 | 47° 33' 50" N; 18° 59' 50" E | 0.346 | -1.9 | 7.0 | 90.2 | 0.01 | 1.9 | 8.5 | |
| 4 | 47° 34' 06" N; 19° 01' 34" E | 0.666 | 4.2 | 7.8 | 53.9 | 0.01 | 18.0 | 5.8 | |
| 5 | 47° 32' 07" N; 19° 01' 03" E | 0.492 | -0.1 | 6.8 | 63.8 | 0.01 | 0.0 | 8.5 | |
| 6 | 47° 31' 33" N; 18° 56' 06" E | 0.220 | -1.9 | 7.4 | 70.4 | 0.01 | 6.0 | 8.5 | |
| 7 | 47° 31' 14" N; 18° 56' 03" E | 0.019 | 2.9 | 7.7 | 62.7 | 0.01 | 13.0 | 7.3 | |
| 8 | 47° 30' 50" N; 18° 56' 34" E | 0.264 | -1.7 | 8.0 | 49.5 | 0.01 | 18.0 | 3.5 | |
| 9 | 47° 30' 53" N; 18° 56' 29" E | 0.059 | 1.9 | 7.5 | 52.8 | 0.01 | 18.0 | 3.8 | |
| 10 | 47° 31' 45" N; 18° 57' 28" E | 0.342 | -1.6 | 7.7 | 62.7 | 0.01 | 5.0 | 6.5 | |
| 11 | 47° 25' 56" N; 18° 59' 22" E | 3.289 | -2.1 | 7.6 | 61.6 | 0.01 | 20.0 | 7.7 | |
| 12 | 47° 30' 57" N; 18° 58' 17" E | 0.407 | -0.8 | 7.3 | 80.3 | 0.01 | 32.0 | 8.5 | |
| 13 | 47° 30' 47" N; 18° 58' 25" E | 0.076 | -1.4 | 7.3 | 59.4 | 0.02 | 20.0 | 8.5 | |
| 14 | 47° 30' 55" N; 18° 58' 55" E | 0.072 | -0.8 | 6.9 | 59.4 | 0.03 | 0.0 | 7.7 | |
| 15 | 47° 31' 45" N; 18° 57' 17" E | 0.394 | -1.1 | 6.3 | 70.4 | 0.05 | 0.0 | 8.5 | |
| 16 | 47° 30' 19" N; 19° 00' 39" E | 0.072 | 0.3 | 7.6 | 60.5 | 0.02 | 30.0 | 6.7 | |
| 17 | 47° 30' 18" N; 19° 00' 41" E | 0.014 | 0.9 | 6.9 | 64.9 | 0.01 | 37.0 | 3.8 | |
| 18 | 47° 29' 12" N; 18° 59' 45" E | 0.032 | 0.7 | 7.5 | 47.3 | 0.01 | 22.0 | 4.0 | |
| 19 | 47° 29' 10" N; 18° 58' 48" E | 0.157 | -1.2 | 6.8 | 80.3 | 0.02 | 0.0 | 8.5 | |
| 20 | 47° 29' 08" N; 18° 58' 59" E | 0.011 | 2.0 | 7.3 | 49.5 | 0.05 | 3.2 | 6.5 | |
| 21 | 47° 30' 05" N; 19° 01' 35" E | 0.258 | 1.3 | 7.5 | 61.6 | 0.05 | 16.0 | 6.5 | |
| 22 | 47° 29' 21" N; 19° 02' 42" E | 0.786 | -0.3 | 7.5 | 68.2 | 0.01 | 38.0 | 8.5 | |
| 23 | 47° 30' 11" N; 18° 57' 50" E | 0.113 | -1.7 | 7.4 | 63.8 | 0.01 | 2.7 | 8.5 | |

^1^ Urbanization index

^2^ Saturation percentage

^3^ Soil organic matter
