# Supplementary material for: Taxonomic and Functional Response of Millipedes (Diplopoda) to Urban Soil Disturbance in a Metropolitan Area
Source: Insects. 2019 Dec 29;11(1):25. doi: 10.3390/insects11010025 (PMC7022796; doi:10.3390/insects11010025)
Supplement: Supplementary file 1 [file insects-11-00025-s001.zip › insects-643986-SUPP/Table_S5.docx]

**Table S5.** Geographical location and habitat characterization (urbanization intensity and topsoil characteristics) of study sites on the Pest side of the Budapest metropolitan area.

| **Site** | **Coordinates** | **Area (km^2^)** | **UI^1^** | **Soil properties** | | | | | |
| --- | --- | --- | --- | --- | --- | --- | --- | --- | --- |
|  |  |  |  | **pH** | **SP^2^ (%)** | **Salt (m/m %)** | **CaCO_3_ (m/m %)** | **SOM^3^ (m/m %)** |  |
| 1 | 47° 30' 36" N; 19° 05' 19" E | 0.316 | -0.8 | 6.6 | 41.8 | 0.06 | 4.9 | 3.1 |  |
| 2 | 47° 29' 31" N; 19° 05' 25" E | 1.424 | 1.7 | 6.9 | 47.3 | 0.04 | 6.0 | 2.2 |  |
| 3 | 47° 28' 54" N; 19° 05' 26" E | 0.258 | 1.8 | 7.2 | 34.1 | 0.01 | 8.4 | 2.5 |  |
| 4 | 47° 28' 50" N; 19° 06' 16" E | 0.408 | -0.1 | 7.0 | 47.3 | 0.01 | 2.1 | 1.4 |  |
| 5 | 47° 30' 10" N; 19° 04' 37" E | 0.064 | 8.0 | 7.1 | 45.1 | 0.01 | 7.5 | 1.9 |  |
| 6 | 47° 25' 04" N; 19° 11' 51" E | 0.609 | -2.1 | 7.1 | 44.4 | 0.01 | 0.9 | 1.5 |  |
| 7 | 47° 26' 37" N; 19° 11' 36" E | 0.366 | 0.2 | 6.8 | 50.6 | 0.08 | 5.7 | 2.5 |  |
| 8 | 47° 27' 35" N; 19° 12' 07" E | 0.669 | -2.1 | 6.9 | 37.4 | 0.01 | 0.2 | 1.2 |  |
| 9 | 47° 28' 28" N; 19° 11' 04" E | 6.019 | -0.3 | 6.9 | 46.2 | 0.03 | 2.6 | 1.9 |  |
| 10 | 47° 28' 55" N; 19° 08' 13" E | 0.067 | 3.1 | 6.8 | 39.6 | 0.01 | 4.0 | 2.5 |  |
| 11 | 47° 28' 49" N; 19° 08' 49" E | 0.275 | 0.8 | 6.5 | 39.6 | 0.01 | 0.3 | 1.2 |  |
| 12 | 47° 29' 37" N; 19° 08' 39" E | 0.476 | 0.6 | 6.9 | 38.5 | 0.01 | 0.9 | 2.1 |  |
| 13 | 47° 26' 26" N; 19° 09' 31" E | 0.377 | 3.0 | 7.0 | 33.0 | 0.01 | 4.1 | 2.4 |  |
| 14 | 47° 35' 09" N; 19° 04' 41" E | 0.839 | -0.6 | 6.9 | 67.1 | 0.09 | 14.4 | 4.2 |  |
| 15 | 47° 34' 56" N; 19° 05' 35" E | 0.819 | 0.8 | 7.1 | 40.7 | 0.01 | 3.8 | 1.1 |  |
| 16 | 47° 35' 05" N; 19° 06' 22" E | 1.326 | -1.5 | 6.8 | 48.4 | 0.01 | 0.9 | 4.0 |  |
| 17 | 47° 32' 39" N; 19° 09' 06" E | 0.942 | -2.0 | 6.7 | 62.7 | 0.03 | 1.7 | 3.4 |  |
| 18 | 47° 32' 34" N; 19° 10' 36" E | 0.407 | -1.9 | 6.9 | 52.8 | 0.01 | 5.0 | 0.6 |  |
| 19 | 47° 30' 26" N; 19° 14' 38" E | 1.551 | -1.6 | 6.3 | 35.2 | 0.01 | 0.1 | 3.0 |  |
| 20 | 47° 24' 16" N; 19° 09' 08" E | 1.028 | -2.1 | 7.2 | 49.5 | 0.04 | 4.2 | 2.3 |  |
| 21 | 47° 25' 38" N; 19° 08' 03" E | 0.516 | 3.7 | 7.1 | 37.4 | 0.01 | 2.7 | 0.7 |  |
| 22 | 47° 24' 48" N; 19° 07' 10" E | 0.480 | -0.4 | 7.2 | 45.1 | 0.05 | 0.3 | 2.0 |  |
| 23 | 47° 37' 29" N; 19° 13' 07" E | 2.594 | -2.1 | 6.7 | 50.6 | 0.04 | 0.2 | 2.1 |  |
| 24 | 47° 34' 18" N; 19° 25' 56" E | 2.324 | -1.9 | 6.7 | 47.3 | 0.05 | 0.7 | 1.3 |  |

^1^ Urbanization index

^2^ Saturation percentage

^3^ Soil organic matter
